# Detecting and describing heterogeneity in health care cost trajectories among asylum seekers

**DOI:** 10.1186/s12913-022-08346-y

**Published:** 2022-07-30

**Authors:** Christina Tzogiou, Jacques Spycher, Raphaël Bize, Javier Sanchis Zozaya, Jeremie Blaser, Brigitte Pahud Vermeulen, Andrea Felappi, Patrick Bodenmann, Joachim Marti

**Affiliations:** 1 Winterthur Institute of Health Economics, Zurich University of Applied Sciences, Gertrudstrasse 15, 8401 Winterthur, Switzerland; 2 Department of Health Sciences and Medicine, University of Lucerne, Lucerne, Switzerland; 3 Department of Epidemiology and Health Systems, Center for Primary Care and Public Health (Unisanté) University of Lausanne, Lausanne, Switzerland; 4 Department of Psychiatry, Lausanne University Hospital (CHUV), Lausanne, Switzerland; 5 General practitioner, Bevaix, Switzerland; 6 Department of Vulnerabilities and Social Medicine, Center for Primary Care and Public Health (Unisanté), Lausanne, Switzerland; 7 Fondation AACTS (Addiction, Community Action, Social Work), Vevey, Switzerland; 8 Department of Vulnerabilities and Social Medicine, Center for Primary Care and Public Health (Unisanté) University of Lausanne, Lausanne, Switzerland

**Keywords:** Asylum seekers, Recipients of emergency aid, Health care cost trajectories, Health care utilization, Switzerland

## Abstract

**Background:**

The mechanism underlying the health care cost trajectories among asylum seekers is not well understood. In the canton of Vaud in Switzerland, a nurse-led health care and medical Network for Migrant Health (“Réseau santé et migration” RESAMI) has established a health care model focusing on the first year after arrival of asylum seekers, called the “community health phase”. This model aims to provide tailored care and facilitate integration into the Swiss health care system. The aim of this study is to explore different health care cost trajectories among asylum seekers during this phase and identify the associated factors.

**Methods:**

We detected different patterns of health care cost trajectories using time-series clustering of longitudinal data of asylum seekers in the canton of Vaud in Switzerland. These data included all adult asylum seekers and recipients of emergency aid who entered the canton between 2012 and 2015 and were followed until 2018. The different clusters of health care cost trajectories were then described using a multinomial logistic regression model.

**Results:**

We identified a concave, an upward trending, and a downward trending cluster of health care cost trajectories with different characteristics being associated with each cluster. The likelihood of being in the concave cluster is positively associated with coming from the Eastern Mediterranean region or Africa rather than Europe and with a higher share of consultations with an interpreter. The likelihood of being in the upward trending cluster, which accrued the highest costs, is positively associated with 20–24-year-olds rather than older individuals, coming from Europe than any other region and having a mental disorder. In contrast to the other two clusters, the likelihood of being in the downward trending cluster is positively associated with having contacted the RESAMI network within the first month after arrival, which might indicate the potential of early intervention. It is also positively associated with older age and living in a group lodge.

**Conclusions:**

Asylum seekers are heterogeneous in terms of health care cost trajectories. Exploring these differences can help point to possible ways to improve the care and supporting services provided to asylum seekers. Our findings could indicate that early and patient-centered interventions might be well-suited to this aim.

**Supplementary Information:**

The online version contains supplementary material available at 10.1186/s12913-022-08346-y.

## Introduction

Asylum seekers frequently live in situations of extreme vulnerability due to migration-related health risks, negative life events and low socio-economic status [1–3]. They also often face communication barriers and lack knowledge about health and the host health care system [4–6]. At the same time, health care providers are often not trained to care for this population, which is often traumatized and has very specific needs [7–10]. Therefore, in addition to providing tailored care to asylum seekers, providing important information on health, prevention and the health care system of the host country can be key and may have positive implications for health, for the efficient use of health care resources and for health care costs [11–13].

In the French-speaking canton of Vaud in Switzerland (covering 10% of the Swiss resident population [14]), a nurse-led Network for Migrant Health (“Réseau santé et migration” RESAMI) of public and private providers was established to provide specifically tailored care to asylum seekers and recipients of emergency aid. The RESAMI network is composed of the Migrant Reception Establishment in the canton of Vaud (“Etablissement Vaudois D'accueil Des Migrants” EVAM), the Migrant Care Unit (”Unité de Soins aux Migrants” USMi), primary care physicians, pharmacies, and specialists. EVAM is a public institution mandated by the canton of Vaud, under the Federal Asylum Act, to register and accommodate asylum seekers and provisionally admitted persons, and to deliver emergency aid to dismissed asylum seekers or those who are in an irregular situation (i.e. “recipients of emergency aid”). The Migrant Care Unit comprises a team of nurses, general practitioners and administrative personnel specialized in the primary care of asylum seekers, and they work in close collaboration with interpreters [15]. While EVAM is responsible for the housing and social care of asylum seekers, the Migrant Care Unit is responsible for delivering and coordinating their health care. It is supported by the other actors of the RESAMI network, such as general practitioners, pediatricians or other specialists, when required (for more information on the responsibilities of each actor see Spycher et al. 2021 [16]).

As soon as asylum seekers and recipients of emergency aid arrive in the canton of Vaud, they are invited to visit one of the facilities of the Migrant Care Unit for health examinations, including immunization. During the first year, called the “community health phase”, they are also encouraged to visit the Migrant Care Unit as a first point of access to health care. With the “community health phase” newly arrived asylum seekers are meant to be integrated into the Swiss health care system. The Migrant Care Unit helps them manage issues related to language and cultural differences, and use efficiently the health care services. The Migrant Care Unit thus acts as a gatekeeper, referring individuals to physicians and specialists within the RESAMI network or the emergency department when needed, and offers follow-up consultations. It also provides them with information on disease prevention and health promotion. As long as they are included in the RESAMI network, individuals can, according to their needs, either continue their care at the Migrant Care Unit or use the “conventional” health care system.

Previous studies have highlighted the need for primary health care among newly arrived asylum seekers [17, 18]. They also have highlighted the importance of identifying and addressing these needs early for preventing somatic and mental health disorders that could even become chronic [19], leading to high health care costs [11, 20, 21]. Bischoff et al. 2009 [22] found that, despite the higher number of diagnosed diseases, younger asylum seekers have lower health care costs compared to local patients, indicating unmet health care needs. Evidence from Germany showed that asylum seekers have more inpatient and emergency department encounters compared to regularly insured people that could have been prevented with better access to primary and preventive care [20]. In fact, asylum seekers demonstrate a lower probability of visiting an outpatient physician with less than 50% of their health care costs being due to outpatient care [5]. Noticeably, costs due to delayed care are shown to be higher than the costs of prevention and health care [11]. In the same vein, a report by the European Union Agency for Fundamental Rights showed that providing preventive care to migrants in an irregular situation compared to providing emergency health care due to delayed access is even cost-saving [21].

Addressing asylum seekers’ complex needs can be challenging from a financial and organizational perspective for the host country, especially considering the heterogeneity among asylum seekers [20, 23, 24]. In order to meet the specific needs of asylum seekers and minimize the burden on the health care system, host authorities need to understand the health care utilization patterns among asylum seekers better. An assessment of the health care cost trajectories can be well-suited to this aim as it goes beyond the number of services utilized and provides information on the evolution of the intensity of care [25]. This is particularly relevant as the timing of health care utilization can have important implications for the health care system, as described above.

In this study, we exploit rich longitudinal data collected by the RESAMI network to identify different trajectories of health care costs among asylum seekers, including recipients of emergency aid in the canton of Vaud. Emergency aid refers to the special residence status of dismissed asylum seekers and not to medical emergency. Moreover, we provide novel information on the characteristics that are associated with the differences between these health care cost trajectories and estimate the statistical relevance of the timing of the first contact with the RESAMI network. As such, we add to the existing literature by shedding new light on the potential mechanisms underlying the trajectories of health care among asylum seekers and recipients of emergency aid.

## Institutional background

Switzerland had in 2020 67,175 recognized refugees granted asylum and 55,638 asylum seekers [26], representing 1.4% of the population. Evidence shows significant differences in health and in health care utilization between immigrants or asylum seekers and non-migrants in Switzerland [27–30].

Despite the broad health care coverage in Switzerland, the health care system is very complex, making it even more difficult for asylum seekers to navigate. It is highly decentralized, including the processing of asylum seekers, which is organized differently in each of the twenty-six cantons [31]. Upon arrival, individuals apply for asylum in one of the six federal asylum centers. If asylum or provisional admission are granted or if the asylum decision procedure is extended, individuals are allocated to one of the cantons according to defined quotas based on the population size of the canton [32]. For the allocation, characteristics such as nationality, intensive support cases^1^ or applicants' request for allocation to a specific canton, for example due to family reunification, are also taken into account. Dismissed asylum seekers can solicit emergency aid from the canton of allocation or from the canton assigned to enforce their departure from Switzerland in case they had not been allocated before the dismissal decision [33].

The mandatory health insurance is covered for asylum seekers by the cantons throughout the entire process, even in the case of dismissal of asylum when the person is under the system of emergency aid [34]. Each canton can buy a different health insurance plan. The canton of Vaud buys for all asylum seekers an insurance plan with a deductible of CHF 2,500 from a pool of private insurers. This coverage includes all essential treatments, medications and devices [35]. Out-of-pocket contributions and traveling costs are also covered by the canton. As a result, asylum seekers do not face any direct financial barriers to accessing health care services, except for additional costs for drugs that are not covered by the insurance. In addition, neither the Federal Act on Health Insurance nor the Asylum Act restrict the benefits to be covered. However, according to Article 82a para. 3 of the Asylum Act, the confederation and the cantons can control the access to the health system by restricting the choice of service providers [36]. Moreover, recipients of emergency aid might face additional barriers due to their administrative status, or due to lack of information regarding their rights on the part of medical professionals outside of the RESAMI network.

## Methods

### Data

The study uses patient-level data collected by the different actors of the RESAMI network. This dataset includes all adult asylum seekers and recipients of emergency aid who entered the canton of Vaud between 2012 and 2015 and were followed until they were granted residence, they exited the country or until the end of the data extraction period on December 31, 2018. However, due to a data linkage problem, 11% of the original sample cannot be used (for more information on the data see [16]). This sample is statistically not significantly different compared to our dataset. Additionally, although this study focuses on the first twelve months, we are also interested in observing how the health care costs evolve in the following eight months after the community health phase. Therefore, we only included individuals in the sample that had had a length of stay of at least twenty months. We do not set the minimum length of stay higher than this so as not to reduce the sample size further.

The dataset provides information on demographic and administrative characteristics (e.g. age, sex, region of origin, language skills, legal status, region of residence, month of arrival, type of housing), health care utilization (e.g. date of treatment, type of service, type of provider, use of interpreter), health care costs^2^ and medication purchases. We aggregated the data by month and divided the health care costs into outpatient, inpatient, other costs, emergency, medication, and cost for the Migrant Care Unit. The costs are in Swiss Francs (CHF, 1 Swiss Franc is as of November 2021 about 1 USD US-Dollar). Diagnostic information was not available in the dataset. However, we were able to identify seven health disorders that are often observed in migrants using medication purchase data: six somatic health disorders (high cholesterol, Type 1 or 2 diabetes, pain/inflammation, hypertension, parasitosis or bacterial infections, tumor such as cutaneous precancer, leukemia, breast cancer or pulmonary cancer) and one group with mental disorders (prescription of antidepressants, anxiolytics, hypnotics, sedatives or antipsychotics). The dosage and frequency of purchase were not considered for this classification.

### Time-series Clustering

We uncovered the different patterns of health care cost trajectories by applying a shape-based time-series clustering algorithm on the longitudinal health care cost data in the first twelve months after arrival. This is a data science technique that is applied to discover patterns and group homogeneous time-series together when there is no former knowledge about clusters [37]. By assessing the health care cost trajectories instead of the accumulated values, we were able to account for the evolution of the intensity of care.

To remove random noise in health insurance data, we first smoothed the trajectories by bins that sum the health care costs of four consecutive months.^3^ This produced three bins for a period of twelve months. There are different smoothing techniques that can be applied, such as fitting each trajectory with unadjusted polynomial regressions. We tested various alternatives and this smoothing strategy seemed to fit the shape of the trajectories the best.

We then also rescaled the trajectories by dividing the value in each bin by the mean of the three bins for each trajectory (i.e. for each individual). We thereby replaced the health care costs with weights relative to the mean costs. This enabled a shape-based comparison between the trajectories that does not take the magnitude of the costs (or any other characteristic) into consideration.

Next, we assigned the asylum seekers into the clusters of homogeneous shapes of health care cost trajectories by using the Euclidean distance for measuring dissimilarity between trajectories and the medoids as the centers of each cluster (i.e. the “centroids”) [38, 39]. This is known as the partition around medoids (PAM) algorithm [40]. With this approach, the centroid is a representative trajectory from the cluster, as it has the smallest average distance to all other trajectories in the same cluster [38, 40]. We opted for the Euclidean distance measure and the PAM algorithm after testing for alternative options. We defined the optimal number of clusters and evaluated the goodness of the clustering structure based on the Silhouette width [41], which is an internal cluster validity index [37]. The Silhouette width can range between -1 and 1 [37, 41].

### Multinomial regression

We assessed the differences between the clusters of health care cost trajectories using multinomial logistic regressions [42] with cluster being the dependent variable. We opted for the multinomial logistic model after testing for the independence of irrelevant alternatives (IIA) assumed in this model [43–45] and for alternative models.

The explanatory variables are time invariant characteristics, namely an indicator of whether the person had at least one consultation at the Migrant Care Unit within the first 30 days after arrival, an indicator of whether the asylum seeker is a recipient of emergency aid, sex, age groups, marital status, region of origin, language skills,^4^ region of residence, an indicator of whether the person is living in a group lodge, an indicator for each of the seven health disorders, the type of health care utilization as the accumulated number of consultations in the first twelve months after arrival (distinguished between outpatient, inpatient, other treatments, emergency and Migrant Care Unit), as well as the share of consultations with an interpreter. The outpatient consultations include treatments in a private practice, hospital, outpatient clinic, and policlinic. Other consultations include, for example, nursing care, treatments with an optician, psychotherapy, and visits for laboratory tests. The aim of controlling for the number of consultations was to assess the composition of health care utilization in each cluster. To test how these consultations influence the estimated associations, we conducted a sensitivity analysis in which these factors were not included in the multivariable model. Furthermore, to make sure that the association of seasonality with the clusters is not included in the estimated coefficients, we also controlled for the month of arrival. For the model specification, the predictor variables were checked for multicollinearity.

To assess the association between the timing of the first contact with the Migrant Care Unit and the likelihood of being in each cluster, we first estimated a univariable model. In a second step, we estimated a multivariable model that fulfilled two purposes. First, the coefficients from the univariable estimation might be biased due to self-selection. With the multivariable estimation, we can control for observed characteristics that might impact this self-selection and assess the change in the coefficients of the timing variable. Second, the multivariable estimation allowed us to identify individual characteristics that are associated with each cluster. Therefore, while the univariable estimation was used to test a hypothesis, the multivariable was used for an exploratory analysis.

The cluster analysis was conducted using R version 4.0.2. All other analyses were conducted using Stata version 16.1.

## Results

### Sample description

After restricting the sample to individuals that had a length of stay of at least twenty months, 2,573 asylum seekers were identified, 14% of whom had been dismissed and therefore required emergency aid ( \* MERGEFORMAT Table 1). One third of the individuals are female, more than half of them are aged between 25 and 39 years old, and 44% come from Africa. Approximately one third of the individuals visited the Migrant Care Unit within the first month. Based on medication bills, 80% had pain or inflammation over a mean observation period of three years, 51% had parasitosis or a bacterial infection, and 42% had a mental disorder. Two-thirds had more than one disorder (e.g. 49% had pain or inflammation and parasitosis or bacterial infection; 40% had pain or inflammation and a mental disorder).

**Table 1:** Descriptive statistics and health care costs per month over the first twelve months

	Proportion in % *n* = 2,573	Monthly health care costs in CHF		
		Mean	Median	IQR
USMi visit 1^st^ month	34.59	1063	623	[358; 1157]
No USMi visit 1^st^ month	65.41	686	415	[245; 745]
Asylum seeker	86.40	828	478	[288; 873]
Emergency aid recipient	13.60	738	430	[249; 889]
Female	31.17	1074	715	[396; 1223]
Male	68.83	699	408	[256; 702]
Age categories				
20 to 24 years	19.43	289	245	[156; 356]
25 to 39 years	56.47	845	514	[319; 939]
40 to 49 years	15.47	1052	626	[388; 1076]
50 to 59 years	4.59	1216	784	[501; 1208]
60 and older	4.04	1587	947	[559; 2009]
Married	30.00	1006	655	[392; 1085]
Not married	70.00	734	415	[252; 750]
Region of origin				
Europe	8.47	1831	989	[519; 1849]
Eastern Mediterranean	39.25	692	490	[289; 840]
Africa	44.35	741	423	[267; 776]
Other	5.91	783	449	[283; 778]
Unknown/stateless	2.02	705	410	[235; 811]
Language				
French	44.97	852	468	[273; 874]
German, Italian or English	13.91	829	479	[289; 805]
Other language	41.12	772	473	[289; 904]
Region of residence				
Haut-Léman	20.60	813	551	[325; 970]
La Côte	13.29	581	372	[226; 645]
Lausanne	48.39	890	463	[272; 889]
Nord Broye	17.72	795	502	[328; 929]
Living in a group lodge	91.22	818	473	[286; 873]
Not living in a group lodge	8.78	791	445	[254; 919]
CHO	4.20	1411	983	[645; 1634]
DM	3.65	1655	989	[592; 1981]
Pain	80.22	894	523	[314; 968]
HYP	8.82	1624	919	[540; 1879]
BAC	50.91	1048	613	[358; 1101]
TUM	0.86	3993	3148	[613; 7131]
PSY	42.01	1147	690	[391; 1224]

Source: own calculations based on data from the RESAMI network

*IQR* Inter-quartile range (distance between the 25th and 75th percentiles), *USMi* Migrant Care Unit, *CHO* High cholesterol, *DM* Type 1 or 2 diabetes, *Pain* Pain/inflammation, *HYP* Hypertension, *BAC* Parasitosis or bacterial infections, *TUM* Tumor, *PSY* Mental disorder

Important differences in mean health care costs in the first twelve months after arrival are observed between the sexes and among age groups ( \* MERGEFORMAT Table 1). Asylum seekers that receive emergency aid have statistically significantly lower mean health care costs than asylum seekers that have been provisionally admitted or granted asylum. Individuals that had a Migrant Care Unit visit within the first month also have lower mean health care costs. A statistically significant difference is also observed between married and unmarried individuals, which is mainly driven by outpatient costs (see Additional file 1). Moreover, the inter-quartile range (IQR) shows that there is high variation in the costs within each group. This can be attributed to the different characteristics of the individuals within the groups, such as access to health care, age and health status.

### Clusters of health care cost trajectories

**Fig. 1:**
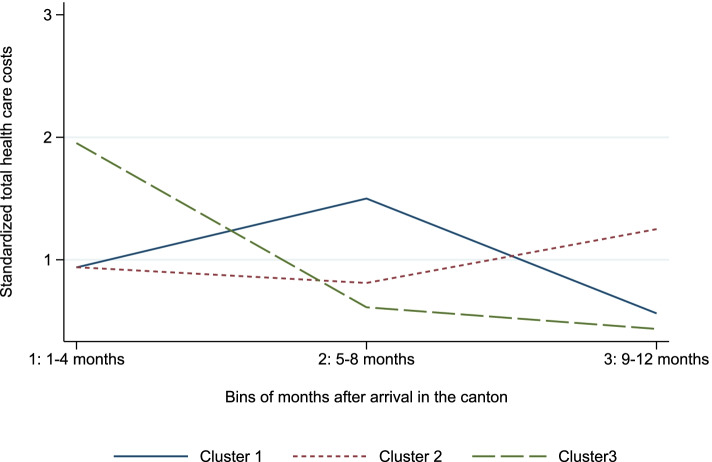
Shape of representative trajectories of each cluster (i.e. centroids)

Based on the shapes of the health care cost trajectories in the first twelve months after arrival in the canton of Vaud and cluster validity criteria, asylum seekers were grouped into three distinct clusters. The Silhouette width was maximized with three clusters equaling 0.36 and showing a higher intra-cluster similarity and lower inter-cluster similarity compared to partitioning the sample to a different number of clusters (Additional file 2). \* MERGEFORMAT Fig. 1 shows the centroids of each cluster during the community health phase. These trajectories are rescaled and therefore only their shapes are relevant for interpretation.^5^ The three clusters were rather homogeneous in size with the first cluster representing 33% of the sample, the second 37% and the third 29%. Fig. S2 in the Additional file 3 shows the health care cost trajectories of ten randomly selected asylum seekers in each cluster to gain an impression of the within cluster variation.

The health care costs in the first cluster increase during the first four months, reaching a peak during the second four-month period and then decreasing sharply. The trajectories of this cluster thus resemble a concave function (concave cluster). In the second cluster, the health care costs are relatively stable between the first and the second four-month periods and then follow an upward trend (upward trending cluster). The health care costs in the third cluster are high during the first four months and then decrease sharply during the following four months followed by a slight downward trend (downward trending cluster).

**Fig. 2:**
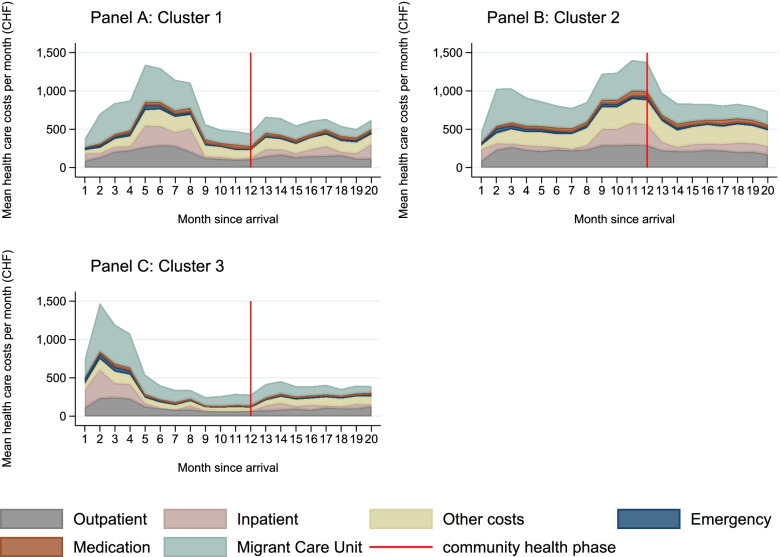
Chronograms of mean monthly health care costs by cluster and type of cost

What differentiates the clusters are the timing and composition of health care costs. \* MERGEFORMAT Fig. 2 shows the mean monthly health care costs decomposed into the six cost components expanded to a period of twenty months. The mean monthly costs over this period are the highest in the upward trending cluster with CHF 931 and the lowest in the downward trending cluster with CHF 519. The observed peaks are mainly driven by inpatient treatments^6^ in all clusters. Other costs also contribute to the peak in the concave cluster (Panel A of \* MERGEFORMAT Fig. 2) and Migrant Care Unit costs to the peak in the downward trending cluster (Panel C of \* MERGEFORMAT Fig. 2). We also observe a generally higher intensity of care in the first months in these clusters, which then decreases. On the other hand, the upward trending cluster (Panel B of \* MERGEFORMAT Fig. 2) exhibits two peaks, with the first being driven by Migrant Care Unit costs and the second by inpatient costs. We also observe higher emergency costs at the beginning in all clusters. This is not surprising, as many asylum seekers have limited knowledge of the local health care system upon their arrival, leading them to seek care in the emergency department [29, 46]. The medication costs decrease in the downward trending cluster after the first six months and remain relatively steady throughout the observation period in the other two clusters. In terms of total health care costs during the twenty-month period,^7^ the upward trending cluster accounts for the highest share with 47%, followed by the concave cluster with 33% and by the downward trending cluster with 21%.

**Fig. 3:**
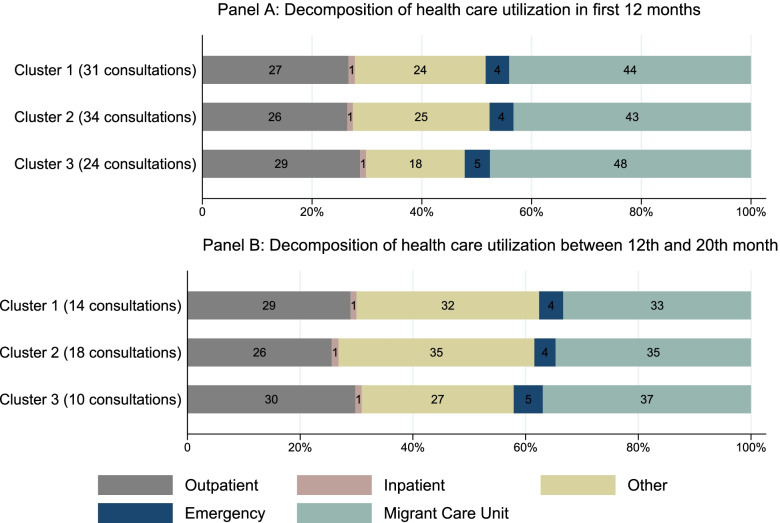
Composition of health care utilization during the community health phase and in the following eight months. Pharmacy visits including the medical costs are not included in this Figure

In line with health care costs, the mean number of consultations is the highest in the upward trending cluster and the lowest in the downward trending cluster ( \* MERGEFORMAT Fig. 3). The decomposition of health care utilization shows that most consultations take place in the Migrant Care Unit ( \* MERGEFORMAT Fig. 3). We also observe a shift from Migrant Care Unit consultation to outpatient and other consultations in all clusters after the community health phase, while the number of emergency and inpatient treatments remain stable.

### Cluster differences

 \* MERGEFORMAT Table 2 shows the average changes in the probability of being in the concave (cluster 1), in the upward-trending (cluster 2) and in the downward-trending cluster (cluster 3) associated with a marginal or discrete unit change in the individual characteristics. The results from the univariable analysis show that asylum seekers that had visited the Migrant Care Unit within the first month have a higher likelihood of being in the downward trending cluster by 17 percentage points and a lower likelihood of being in the concave cluster. After adjusting for all other factors, having visited the Migrant Care Unit within the first month remained positively associated with the downward trending cluster and negatively associated with the concave cluster and became also negatively associated with the upward trending cluster. This is also reflected in the composition of the costs in the chronograms ( \* MERGEFORMAT Fig. 2).

**Table 2:** Factors associated with each cluster

	Univariable Model			Multivariable Model		
	Cluster 1	Cluster 2	Cluster 3	Cluster 1	Cluster 2	Cluster 3
USMi visit 1st month	-0.137***	-0.0361	0.173***	-0.136***	-0.084***	0.219***
	(-0.174 to -0.101)	(-0.075 to 0.003)	(0.135 to 0.211)	(-0.173 to -0.098)	(-0.122 to -0.045)	(0.181 to 0.257)
Emergency aid recipient				0.013	-0.063*	0.050
				(-0.044 to 0.070)	(-0.117 to -0.008)	(-0.002 to 0.101)
Female				0.014	-0.054*	0.040
				(-0.030 to 0.058)	(-0.097 to -0.010)	(-0.001 to 0.082)
Age categories (ref: 20 to 24 years)						
25 to 39 years				-0.016	-0.083**	0.099***
				(-0.074 to 0.042)	(-0.143 to -0.024)	(0.055 to 0.144)
40 to 49 years				-0.060	-0.077*	0.137***
				(-0.133 to 0.013)	(-0.153 to -0.001)	(0.074 to 0.200)
50 to 59 years				-0.043	-0.077	0.120*
				(-0.147 to 0.062)	(-0.184 to 0.030)	(0.019 to 0.220)
60 and older				-0.104	-0.140*	0.245***
				(-0.211 to 0.003)	(-0.252 to -0.029)	(0.125 to 0.364)
Married				0.043	-0.030	-0.013
				(-0.005 to 0.091)	(-0.077 to 0.017)	(-0.057 to 0.030)
Region of origin (ref: Europe)						
Eastern Mediterranean				0.111**	-0.150***	0.038
				(0.044 to 0.179)	(-0.229 to -0.071)	(-0.030 to 0.107)
Africa				0.113**	-0.140***	0.027
				(0.045 to 0.182)	(-0.221 to -0.060)	(-0.042 to 0.096)
Other				0.076	-0.153**	0.077
				(-0.018 to 0.170)	(-0.258 to -0.049)	(-0.017 to 0.172)
Unknown/stateless				0.118	-0.155*	0.037
				(-0.024 to 0.261)	(-0.304 to -0.006)	(-0.098 to 0.172)
Language skills (ref: French)						
German, Italian or English				0.018	-0.037	0.019
				(-0.021 to 0.057)	(-0.077 to 0.003)	(-0.018 to 0.056)
Other language				-0.006	0.025	-0.020
				(-0.062 to 0.051)	(-0.033 to 0.084)	(-0.070 to 0.031)
Residence region (ref: Lausanne)						
Haut-Léman				0.029	-0.005	-0.024
				(-0.022 to 0.079)	(-0.055 to 0.045)	(-0.070 to 0.022)
La Côte				0.010	0.021	-0.031
				(-0.048 to 0.068)	(-0.038 to 0.080)	(-0.084 to 0.022)
Nord Broye				-0.020	0.048	-0.027
				(-0.072 to 0.031)	(-0.006 to 0.102)	(-0.075 to 0.020)
Group lodge				-0.054	-0.039	0.093**
				(-0.128 to 0.020)	(-0.115 to 0.037)	(0.035 to 0.151)
CHO				0.083	0.016	-0.099*
				(-0.028 to 0.194)	(-0.091 to 0.124)	(-0.186 to -0.013)
DB				0.042	-0.077	0.035
				(-0.072 to 0.157)	(-0.179 to 0.024)	(-0.078 to 0.148)
Pain				0.045	-0.056*	0.011
				(-0.004 to 0.095)	(-0.109 to -0.003)	(-0.033 to 0.055)
HYP				-0.071*	0.033	0.037
				(-0.140 to -0.001)	(-0.042 to 0.109)	(-0.037 to 0.111)
BAC				-0.009	0.009	-0.000
				(-0.050 to 0.032)	(-0.033 to 0.051)	(-0.038 to 0.038)
TUM				-0.166	-0.026	0.192
				(-0.337 to 0.006)	(-0.218 to 0.165)	(-0.013 to 0.397)
PSY				-0.014	0.045*	-0.031
				(-0.055 to 0.027)	(0.003 to 0.087)	(-0.069 to 0.007)
Type of treatment						
Number of outpatient consultations				0.001	0.005*	-0.006**
				(-0.003 to 0.005)	(0.001 to 0.010)	(-0.010 to -0.002)
Number of inpatient consultations				0.014	-0.037***	0.023*
				(-0.005 to 0.034)	(-0.059 to -0.015)	(0.003 to 0.043)
Number of other consultations				0.004*	0.006***	-0.010***
				(0.001 to 0.007)	(0.003 to 0.009)	(-0.014 to -0.006)
Number of emergency consultations				-0.005	0.004	0.001
				(-0.015 to 0.004)	(-0.005 to 0.014)	(-0.009 to 0.011)
Number of USMi consultations				0.003**	0.005***	-0.009***
				(0.001 to 0.006)	(0.003 to 0.008)	(-0.012 to -0.006)
Share of consultations with interpreter				0.144**	-0.036	-0.108*
				(0.052 to 0.235)	(-0.132 to 0.061)	(-0.198 to -0.018)
Seasonality	No			Yes		
Number of observations	2573			2573		
Log likelihood	-2769			-2583		

Source: own calculations based on data from the RESAMI network

The Table shows the shows the average changes in the probability of being in the concave (cluster 1), in the upward-trending (cluster 2) and in the downward-trending cluster (cluster 3) associated with a marginal or discrete unit change in the individual characteristics. 95% confidence intervals in parentheses. * *p* < 0.05 ** *p* < 0.01 *** *p* < 0.001

*Ref* Reference group, *USMi* Migrant Care Unit, *CHO* High cholesterol, *DM* Type 1 or 2 diabetes, *Pain* Pain/inflammation, *HYP* Hypertension, *BAC* Parasitosis or bacterial infections, *TUM* Tumor, *PSY* Mental disorder

The number of consultations and share of consultations with an interpreter refer to the first twelve months

The concave cluster is more likely to include individuals that come from the Eastern Mediterranean region or Africa compared to individuals that come from Europe, while it is less likely to include individuals with hypertension. The accumulated number of consultations at twelve months also shows that the likelihood of being in this cluster increases with the number of other or Migrant Care Unit consultations. An increase in the share of consultations with an interpreter also increases the likelihood of being in this cluster.

The upward trending cluster is more likely to include younger individuals aged 20–24 years compared to older age groups (besides those aged 50–59 years), asylum seekers from Europe compared to those of any other region and individuals with mental health conditions. By contrast, it is less likely to include recipients of emergency aid compared to other asylum seekers, females and individuals with pain or an inflammation. Additionally, a higher number of outpatient, other or Migrant Care Unit consultations increase the likelihood of being in this cluster, while a higher number of inpatient treatments decrease this likelihood.

The downward trending cluster is more likely to include older individuals compared to 20–24-year-olds and individuals that live in a group lodge compared to individuals that have a private lease or live in individual accommodation. Both findings are noteworthy considering that age [47] and living in institutionalized accommodation [11] are shown to be positively associated with health care costs and worse health outcomes. By contrast, this cluster is less likely to include individuals with high cholesterol. Additionally, a higher number of inpatient consultations increase the likelihood of being in this cluster, while a higher number of outpatient, other or Migrant Care Unit consultations decrease the likelihood of being in this cluster. This corresponds to the dynamic observed in \* MERGEFORMAT Fig. 2 and the lower health care utilization observed in \* MERGEFORMAT Fig. 3. An increase in the share of consultations with an interpreter also decreases the likelihood of being in this cluster.

Sensitivity analyses related to the health care utilization variables also showed similar results. The most noticeable differences were the insignificant coefficients of age groups and the fact that individuals with a mental disorder were statistically significantly less likely to be in the downward trending cluster. This association is probably gauged by the number of other consultations, which include psychotherapies, in the multivariable model shown in \* MERGEFORMAT Table 2.

## Discussion

### Identification of main shapes of health care cost trajectories

In this study, we identified three main shapes of health care cost trajectories among asylum seekers or recipients of emergency aid during the first twelve months after their arrival. The upward trending cluster accounted for the highest share of the total health care costs (47%), followed by the concave cluster (33%) and by the downward trending cluster (21%). These shapes might reflect differences in demographic characteristics, health care needs and health care access among asylum seekers.

### Possible reasons for downward trending health care cost trajectories

We showed that individuals that had visited the Migrant Care Unit within the first month were more likely to have a downward trending health care cost trajectory. This trajectory is characterized by higher intensity of care in the first months mainly driven by inpatient and Migrant Care Unit costs, while Migrant Care Unit visits comprised the highest share of utilized services. This could reflect two cases. An early visit to the Migrant Care Unit could, on the one hand, lead to the detection and treatment of (pre-existing) health conditions, and subsequently to high costs in the first months that would otherwise have accrued later. On the other hand, it could lead to the (primary or secondary) prevention of health problems, thus preventing future costs [13, 21, 48]. The latter case could, therefore, indicate the potential of earlier medical interventions in the host country, and in particular Migrant Care Unit visits, for improving health and reducing costs in the longer term. It is, however, important to note that we cannot identify the precise sequence of health care utilization in the different settings when multiple treatments happened on the same day. We can, therefore, not infer anything about the precise contribution of RESAMI to this shape. Lower health care costs could also be explained by the fact that recipients of emergency aid are more likely to be in that cluster. As shown in \* MERGEFORMAT Table 1, these asylum seekers exhibit lower health care costs compared to asylum seekers without emergency aid. This might be due to lower health care utilization because, on the one hand, they might not know their rights to health care and, for example, fear for their residence status [49, 50]; on the other hand, they might face more barriers to accessing health care outside of the RESAMI network. Although this association is statistically significant only at 10% significance level, the lower level of costs in this cluster could be related to poorer access to health care.

### Possible reasons for upward trending health care cost trajectories

A noticeable factor of the upward trending cluster, which is described by high health care costs that persist throughout the twenty-month period, is that it is more likely to include people with a mental disorder. Along the same lines, Maier et al. 2010 [51] have found significant differences in the health care costs between individuals with and without a psychiatric treatment. This is especially relevant for policy makers considering the high prevalence of mental disorders among asylum seekers [52], which often remain undiscovered due to lack of screening [4] or language barriers [30, 53]. Evidence from Switzerland shows that despite a prevalence of mental disorders at 41%, the share of asylum seekers receiving psychiatric treatment is at 26% [51]. This is also relevant considering the poor and unstable course of mental health in traumatized asylum seekers and refugees [54, 55] that could become chronic without early intervention [19]. This dynamic could correspond to the sudden peak in the health care costs observed in Panel B of \* MERGEFORMAT Fig. 2 during the last quarter of the community health phase. Despite some improvements in mental health after arrival [54, 55], two recent systematic reviews [3, 56] have shown that length of asylum process, post-migration stress, and discrimination can lead to psychiatric disorders. The high level of costs in the upward trending cluster could also reflect the fact that recipients of emergency aid are less likely to be in this cluster compared to the other asylum seekers, who exhibit higher health care costs.

### Role of region of origin

In line with previous findings [11, 20, 22], we also found that origin is statistically relevant in distinguishing between the different shapes of health care cost trajectories. Individuals coming from the Eastern Mediterranean region or Africa are more likely to have a concave health care cost trajectory than individuals coming from Europe, while the latter are more likely to have an upward trending health care cost trajectory compared to all other regions. This could reflect differences in cultural norms or expectations from the health care system. It could also reflect regional differences in health care needs. Bauhoff et al. 2018 [20] show, for example, that asylum seekers from the western Balkan states have a higher risk for mental and behavioral disorders than asylum seekers from other regions.

### Limitations

Despite our novel analysis and findings, there are some limitations. First, the observed health care cost trajectories might not represent asylum seekers in cantons or countries without a patient-centered nurse-led health care network such as the one offered in the canton of Vaud. The possible impact of such a network on the health care cost trajectories could be evident in our study from the association between the timing of the first contact with the Migrant Care Unit and the likelihood of being in each cluster. However, the observed associations cannot be interpreted as causal due to the cross-sectional design used in the multinomial analysis. This constitutes the second limitation of our study. A possible way to identify causal relationships would be by comparing the canton of Vaud with a synthetic control group created on the basis of the other Swiss cantons that do not have such a nurse-led primary care system for asylum seekers. Third, despite the careful assessment of medication purchase, the identification of the disorders included subjective decisions that could induce measurement errors and bias. For example, some disorders can be treated without medication, and won’t be identified with the present methodology, while some medication can have different indications and can thus be used for different diseases. In this case, the most probable diagnostic category was selected, but we cannot know with certainty that the patient presented a particular disorder. Fourth, due to the data linkage problem, the sample might not fully represent all asylum seekers in the canton of Vaud. In addition, the share of individuals speaking French in our sample is higher than the share in the full sample of asylum seekers in the canton of Vaud described in Spycher et al. 2021 [16]. This indicates that our sample might be rather selective. Our sample includes only those who had a length of stay in the canton of Vaud of at least twenty months. This could thus imply that asylum seekers are more likely to remain in the country if they can speak the local language. This is a hypothesis that cannot be tested in this study.

### Comparison to previous studies

Few studies have assessed the health care costs or their trajectories among asylum seekers and the potential of early care. These studies take different approaches to ours by providing valuable information on the aggregated trajectories of asylum seekers [16] or the effect of health care access restrictions on health care costs [11, 20, 21]. To the best of our knowledge, our study is the first to assess the different patterns of health care utilization on a monthly basis and their associated factors, and explore the potential of early interventions.

### Possible mechanisms and policy implications

From a policy perspective, our study could thus be particularly interesting, as it points to possible ways policy makers could improve the care and supporting services provided to asylum seekers and in turn improve asylum seekers’ health care cost trajectories. For example, the shape of the downward trending cluster could indicate that early interventions might have positive implications on asylum seekers’ future health care costs. The positive association of the timing of the first contact with the Migrant Care Unit of the RESAMI network with the downward trending cluster, could also indicate that a nurse-led health care network might be well-suited to providing these interventions. Nurse-led primary care systems have been shown to provide appropriate treatment to asylum seekers [15, 57, 58]. A previous study [16] assessing this health care model in the canton of Vaud found that asylum seekers visited the Migrant Care Unit more often during the community health phase and then shifted to the conventional health care system. According to the authors, this indicates the great need for health care in newly arrived asylum seekers and the effectiveness of the community health phase in enabling the individuals to navigate the health care system autonomously.

Moreover, the positive association of the upward trending cluster with mental health shows the association between mental health and high health care costs. At the same time, the fact that people with higher cost trajectories are more likely to not have visited the Migrant Care Unit within the first month could indicate the lost potential of the nurse-led health care network in providing appropriate health care to asylum seekers. This is especially relevant for mental health disorders considering its high prevalence in this population and the importance of the timing of offering culture-sensitive treatments [19]. Although the nurses of the Migrant Care Unit do not provide mental health care themselves, they can identify mental health problems and refer the individuals to specialists within the RESAMI network, who are also specialized in the treatment of asylum seekers.

Therefore, this study indicates that a patient-centered nurse-led health care network could be well suited to offering early care adapted to asylum seekers’ specific health care needs. Nevertheless, further research that analyzes data on diagnosis, treatment, compliance with care, and health outcomes in regions with and without a nurse-led health care system should be conducted to gain a better understanding of the drivers of the shape of health care cost trajectories.

## Conclusions

We show that asylum seekers compose a heterogeneous group of people in terms of health care cost trajectories and provide first evidence on the characteristics that could distinguish these differences. Our findings could indicate that early and patient-centered interventions might be well-suited to providing appropriate health care to asylum seekers.

## Supplementary Information

**Additional file 1:** **Fig. S1.** Silhouette width by number of clusters.

**Additional file 2:** **Table S1.** Descriptive statistics by cost category.

**Additional file 3:** **Fig. S2**. Shape of a random draw of trajectories per cluster.

## Data Availability

The data that support the findings of this study are available from the RESAMI network but restrictions apply to the availability of these data which were used under license for the current study and therefore are not publicly available. However, data are available from the authors upon reasonable request and with permission of the RESAMI network.

## Abbreviations

RESAMI: Réseau santé et migration [Network for Migrant Health]
EVAM: Etablissement Vaudois D'accueil Des Migrants [Migrant Reception Establishment in the canton of Vaud]
USMi: Unité de Soins aux Migrants [Migrant Care Unit]
CHF: Swiss Francs
IIA: Independence of irrelevant alternatives
Ref: Reference group
CHO: High cholesterol
DM: Type 1 or 2 diabetes
Pain: Pain/inflammation
HYP: Hypertension
BAC: Parasitosis or bacterial infections
TUM: Tumor
PSY: Mental disorder

## Footnotes

1 Intensive support cases are, for example, asylum seekers with health problems or unaccompanied minors.

2 With costs, we refer to the billed charges that might not cover the full costs due to out of pocket payments.

3 The dates of the health care costs refer to the treatment and not the billing dates.

4 Language skills were judged by the administrative personnel of EVAM.

5 The same cluster may include individuals with very high or very low health care costs but with similar shape of trajectory.

6 Inpatient treatments cannot be distinguished between preference-sensitive and preference-independent.

7 The same applies when the health care costs during the first twelve months are considered.
