# Supplementary figures and images for: Detecting and describing heterogeneity in health care cost trajectories among asylum seekers
Source: BMC Health Serv Res. 2022 Jul 30;22:978. doi: 10.1186/s12913-022-08346-y (PMC9339203; doi:10.1186/s12913-022-08346-y)

## Additional file 1

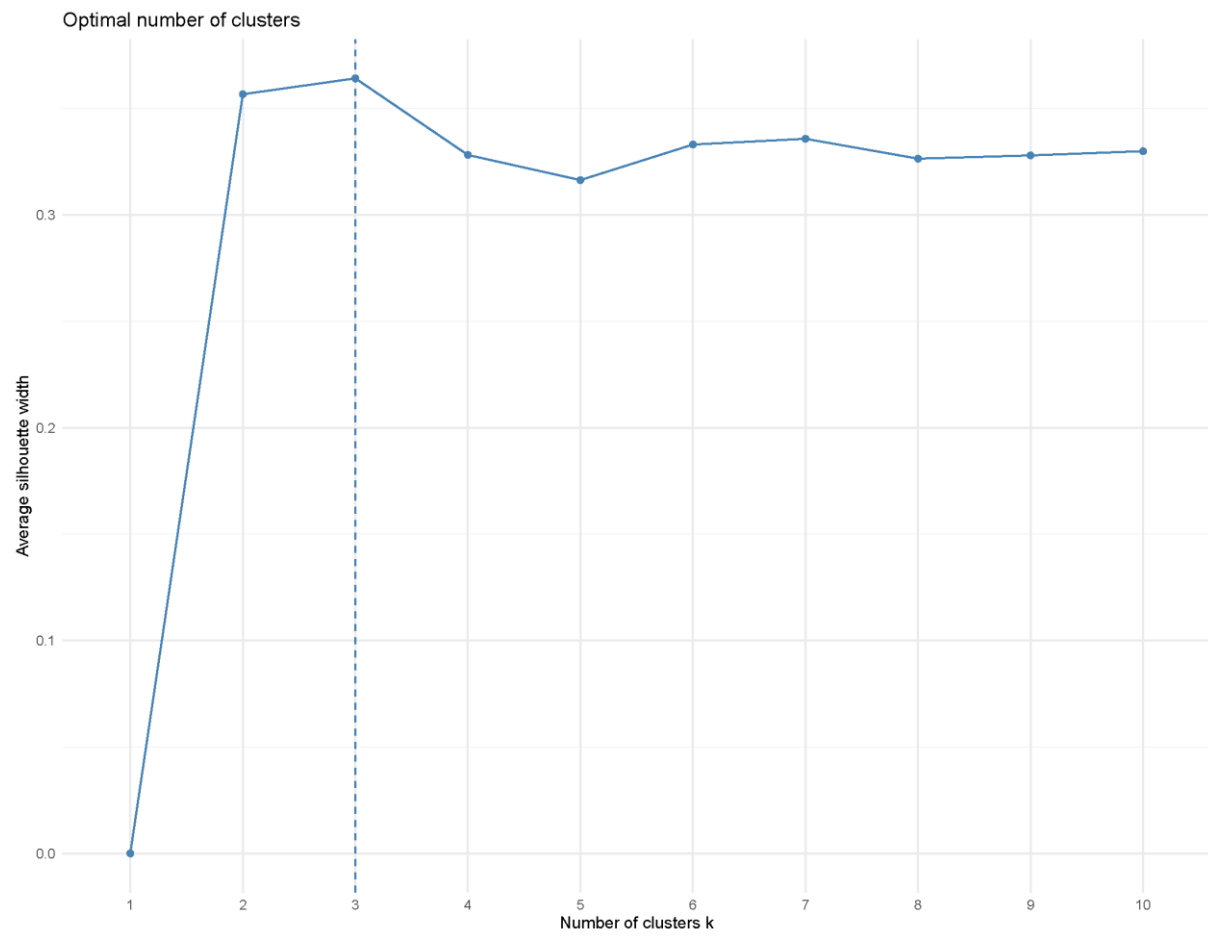

**Fig. S1** Silhouette width by number of clusters

Supplement: Supplementary file 1 — Additional file 1: Fig. S1. Silhouette width by number of clusters. [file 12913_2022_8346_MOESM1_ESM.pdf]
