# Supplementary material for: Detecting and describing heterogeneity in health care cost trajectories among asylum seekers
Source: BMC Health Serv Res. 2022 Jul 30;22:978. doi: 10.1186/s12913-022-08346-y (PMC9339203; doi:10.1186/s12913-022-08346-y)
Supplement: Supplementary file 2 — Additional file 2: Table S1. Descriptive statistics by cost category. [file 12913_2022_8346_MOESM2_ESM.pdf]

Additional file 2

Table S1 Descriptive statistics by cost category

	Monthly health care cost in CHF by cost category:																	
	Outpatient			Hospital			Other			Medication			Emergency			USMi		
	Mean	Median	IQR	Mean	Median	IQR	Mean	Median	IQR	Mean	Median	IQR	Mean	Median	IQR	Mean	Median	IQR
USMi visit 1st month	263	120	[34; 271]	151	0	[0; 0]	198	60	[5; 203]	56	17	[3; 47]	41	3	[0;54]	354	289	[223; 401]
No USMi visit 1st month	147	66	[16; 171]	92	0	[0; 0]	117	31	[0; 122]	31	9	[0; 30]	32	0	[0; 38]	267	223	[156; 334]
Asylum seeker	189	80	[22; 204]	116	0	[0; 0]	148	43	[3; 148]	40	12	[1; 36]	35	0	[0; 45]	301	245	[178; 356]
Emergency aid recipient	178	79	[22; 236]	87	0	[0; 0]	128	21	[0; 124]	42	8	[0; 35]	33	0	[0; 39]	271	223	[134; 334]
Female	263	159	[60; 301]	148	0	[0; 71]	223	91	[24; 247]	55	23	[6; 53]	46	11	[0; 60]	339	267	[178; 423]
Male	153	56	[10; 155]	96	0	[0; 0]	110	23	[0; 103]	33	8	[0; 27]	30	0	[0; 36]	277	245	[156; 334]
Age categories																		
20 to 24 years	4	0	[0; 0]	2	0	[0; 0]	6	0	[0; 0]	1	0	[0; 0]	1	0	[0; 0]	276	245	[156; 334]
25 to 39 years	191	97	[42; 215]	134	0	[0; 0]	149	55	[14; 154]	35	14	[5; 35]	42	13	[0; 56]	294	245	[178; 356]
40 to 49 years	289	152	[63; 300]	134	0	[0; 0]	219	82	[25; 215]	66	23	[8; 57]	43	13	[0; 52]	301	267	[156; 378]
50 to 59 years	357	186	[109; 336]	95	0	[0; 0]	279	143	[60;301]	84	40	[19; 93]	40	5	[0; 47]	362	245	[178; 423]
60 and older	440	268	[171; 440]	274	0	[0; 117]	324	136	[67; 248]	146	72	[36; 133]	63	31	[0; 98]	340	212	[78; 456]
Married	272	146	[65; 293]	143	0	[0; 0]	196	82	[26; 228]	55	26	[8; 61]	42	13	[0; 56]	297	245	[156; 356]
Not married	151	56	[0; 165]	99	0	[0; 0]	123	23	[0; 111]	33	7	[0; 26]	32	0	[0; 37]	297	245	[178; 356]
Region of origin																		
Europe	527	227	[87; 402]	423	0	[0; 276]	384	200	[45; 490]	95	39	[12; 91]	57	16	[0; 68]	345	278	[156; 423]
Eastern Mediterranean	152	89	[20; 193]	63	0	[0; 0]	122	51	[3; 148]	35	14	[0; 42]	37	0	[0; 46]	283	245	[156; 356]
Africa	155	60	[13; 180]	96	0	[0; 0]	121	24	[0; 106]	36	8	[0; 23]	31	0	[0; 39]	302	245	[178; 356]
Other	201	75	[34; 187]	122	0	[0; 0]	142	38	[7; 110]	25	11	[2; 33]	22	0	[0; 24]	271	223	[178; 312]
Unknown/stateless	114	63	[6; 143]	71	0	[0; 0]	140	24	[0; 98]	26	10	[0; 28]	32	0	[0; 49]	322	256	[156; 334]
Language																		
French	201	73	[0; 201]	126	0	[0; 0]	154	41	[0; 155]	38	11	[0; 35]	36	0	[0; 46]	296	245	[156; 356]

German Italian or English	184	80	[31; 199]	118	0	[0; 0]	142	29	[4; 124]	41	10	[1; 31]	34	7	[0; 44]	310	245	[178; 378]
Other language	173	87	[28; 213]	95	0	[0; 0]	137	44	[4; 138]	41	12	[2; 37]	34	0	[0; 41]	293	245	[156; 356]
Region of residence																		
Haut-Léman	156	102	[36; 216]	101	0	[0; 0]	164	63	[8; 178]	45	19	[3; 51]	37	0	[0; 45]	310	267	[178; 378]
La Côte	75	33	[0; 73]	71	0	[0; 0]	96	15	[0; 94]	17	5	[0; 18]	26	0	[0; 36]	297	245	[156; 378]
Lausanne	243	98	[22; 246]	135	0	[0; 0]	157	40	[0; 142]	47	10	[0; 34]	34	0	[0; 42]	274	223	[156; 378]
Nord Broye	155	78	[29; 192]	93	0	[0; 0]	129	41	[4; 142]	32	13	[2; 39]	41	8	[0; 53]	345	289	[200; 423]
Living in a group lodge	181	76	[20; 197]	109	0	[0; 0]	144	37	[0; 140]	38	11	[0; 34]	34	0	[0; 43]	312	267	[178; 378]
Not living in a group lodge	253	130	[46; 303]	150	0	[0; 0]	158	72	[9; 170]	56	21	[4; 69]	39	0	[0; 53]	135	111	[67; 156]
CHO	400	230	[138; 345]	167	0	[0; 97]	251	144	[82; 306]	106	82	[34; 127]	56	22	[0; 78]	430	334	[200; 456]
DB	383	237	[124; 383]	249	0	[0; 149]	330	141	[60; 321]	152	95	[37; 193]	56	20	[0; 79]	485	367	[156; 646]
Pain	207	96	[29; 226]	126	0	[0; 0]	164	51	[4; 165]	43	14	[2; 40]	39	7	[0; 51]	314	267	[178; 378]
HYP	510	229	[128; 406]	238	0	[0; 130]	313	135	[45; 343]	111	62	[20; 122]	60	23	[0; 87]	391	289	[178; 445]
BAC	255	124	[36; 276]	168	0	[0; 0]	191	66	[7; 197]	54	22	[5; 54]	47	12	[0; 61]	333	267	[178; 401]
TUM	1530	708	[242; 1494]	598	0	[0; 530]	897	415	[44; 1654]	408	41	[19; 250]	78	63	[0; 106]	482	301	[223; 601]
PSY	271	130	[39; 284]	183	0	[0; 0]	225	86	[13; 265]	56	24	[5; 58]	49	13	[0; 65]	362	312	[200; 445]

Source: own calculations based on data from the RESAMI network.

Notes: Costs refer to the mean costs per month over the first twelve months after arrival, IQR: inter-quartile range (distance between the 25th and 75th percentiles), USMi: Migrant Care Unit, CHO: high cholesterol, DM: Type 1 or 2 diabetes, Pain: pain/inflammation, HYP: hypertension, BAC: parasitosis or bacterial infections, TUM: tumor, PSY: mental disorder.
