# Supplementary material for: Detecting and describing heterogeneity in health care cost trajectories among asylum seekers
Source: BMC Health Serv Res. 2022 Jul 30;22:978. doi: 10.1186/s12913-022-08346-y (PMC9339203; doi:10.1186/s12913-022-08346-y)
Supplement: Supplementary file 3 — Additional file 3: Fig. S2. Shape of a random draw of trajectories per cluster. [file 12913_2022_8346_MOESM3_ESM.pdf]

Additional file 3

Panel A: Cluster 1

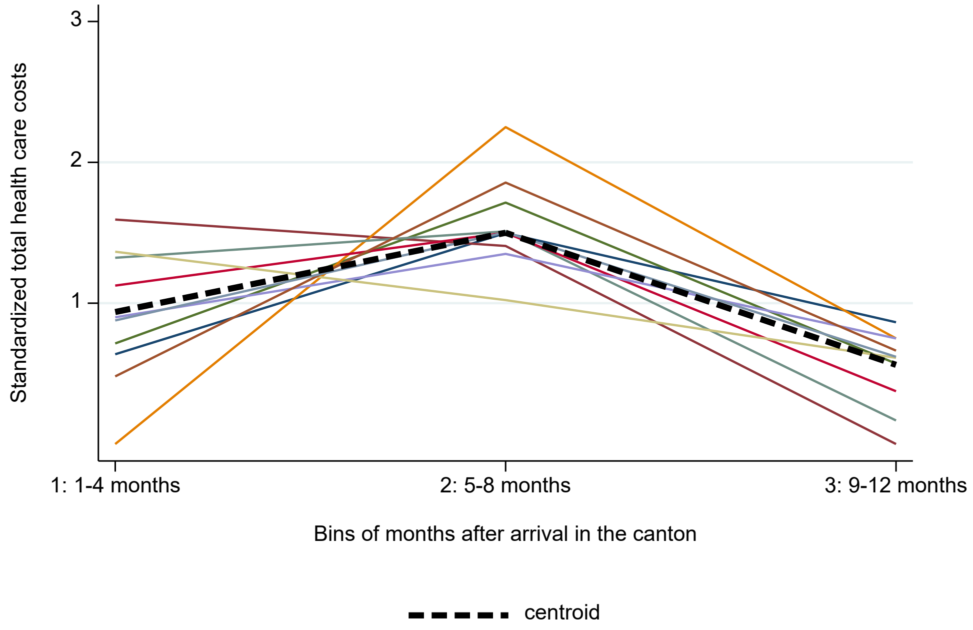

Panel B: Cluster 2

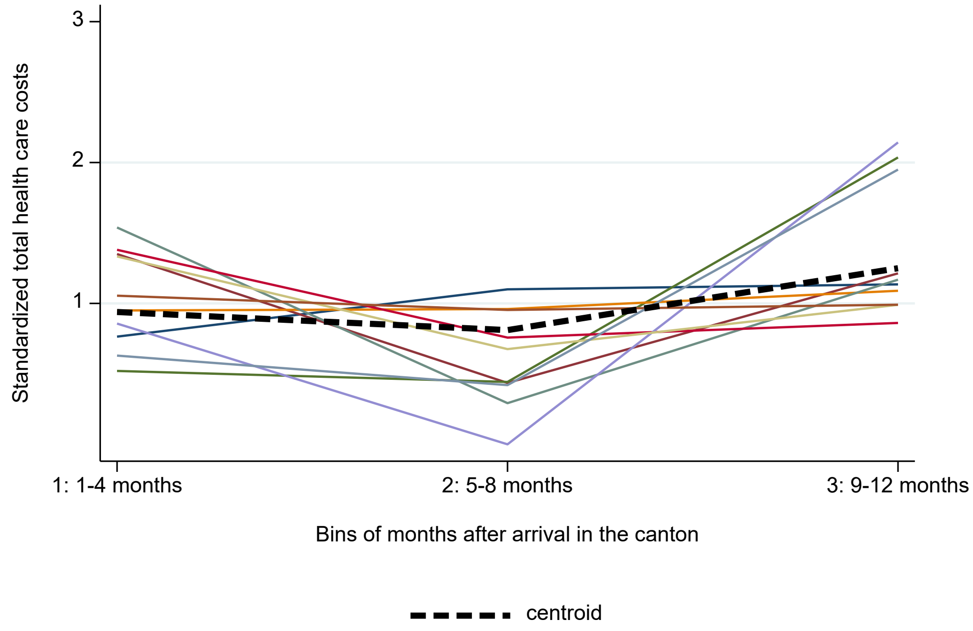

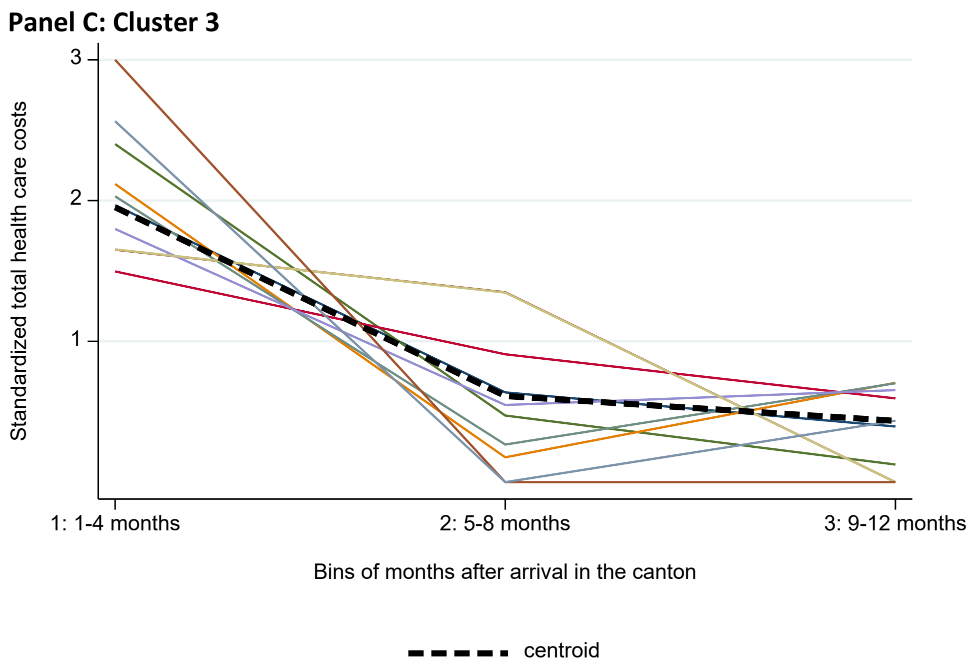

Fig. S2 Shape of a random draw of trajectories per cluster
